# Unwinding the twister ribozyme: from structure to mechanism

**DOI:** 10.1002/wrna.1402

**Published:** 2016-11-14

**Authors:** Jennifer Gebetsberger, Ronald Micura

**Affiliations:** ^1^ Institute of Organic ChemistryLeopold‐Franzens University and Center of Molecular Biosciences Innsbruck CMBIInnsbruckAustria

## Abstract

The twister ribozyme motif has been identified by bioinformatic means very recently. Currently, four crystal structures with ordered active sites together with a series of chemical and biochemical data provide insights into how this RNA accomplishes its efficient self‐cleavage. Of particular interest for a mechanistic proposal are structural distinctions observed in the active sites that concern the conformation of the U‐A cleavage site dinucleotide (in‐line alignment of the attacking 2′‐O nucleophile to the to‐be‐cleaved P—O5′ bond versus suboptimal alignments) as well as the presence/absence of Mg^2+^ ions at the scissile phosphate. All structures support the notion that an active site guanine and the conserved adenine at the cleavage site are important contributors to cleavage chemistry, likely being involved in general acid base catalysis. Evidence for innersphere coordination of a Mg^2+^ ion to the pro‐S nonbridging oxygen of the scissile phosphate stems from two of the four crystal structures. Together with the finding of thio/rescue effects for phosphorothioate substrates, this suggests the participation of divalent ions in the overall catalytic strategy employed by twister ribozymes. In this context, it is notable that twister retains wild‐type activity when the phylogenetically conserved stem P1 is deleted, able to cleave a single nucleotide only. *WIREs RNA* 2017, 8:e1402. doi: 10.1002/wrna.1402

For further resources related to this article, please visit the WIREs website.

## INTRODUCTION

**Figure 1:**
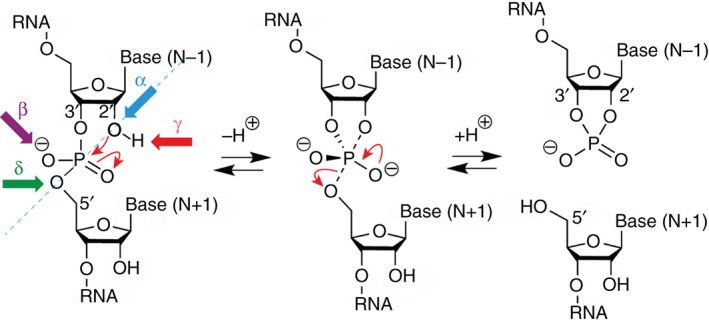
RNA phosphodiester cleavage by phosphoester transfer involving the 2′‐hydroxyl group. The internucleotide linkage (‘scissile’ phosphate)^17^ passes through a pentacoordinate transition state that results in two cleavage products carrying either a 2′,3′‐cyclic phosphate terminus or a 5′‐hydroxyl terminus. The four catalytic strategies that can impact on the reaction are: α, in‐line nucleophilic attack, S_N_2‐type (blue); β, neutralization of the (developing) negative charge on nonbridging phosphate oxygens (purple); γ, deprotonation of the 2′‐hydroxyl group (red); and δ, neutralization of negative charge on the 5′‐oxygen atom by protonation (green).

Since the first reports on RNA strand scission in the early 1950s^1^, ^2^, ^3^, ^4^, ^5^ and the discovery of the first ribozymes in the 1980s,^6^, ^7^ numerous chemical, biochemical, and biophysical studies have been performed, that nowadays can be compared and contrasted with the corresponding crystal structures.^8^, ^9^, ^10^, ^11^, ^12^, ^13^, ^14^, ^15^, ^16^ Therefore, our understanding has already enormously improved on how small self‐cleaving (‘nucleolytic’) ribozymes are able to perform the same internal transesterification reaction although possessing different and unique folds and active sites (Box 1). We know now that small self‐cleaving ribozymes employ diverse strategies to catalyze their site‐specific phosphodiester cleavage, a reaction of the phosphodiester unit with its adjacent ribose 2′‐hydroxyl group, resulting in two RNA fragments that carry 2′,3′‐cyclic phosphate and 5′‐hydroxyl termini, respectively (Figure 1).^18^, ^19^ Cleavage by nucleolytic ribozymes further implies that this reaction proceeds via a penta‐coordinated phosphorane species that is favorably achieved through a S_N_2‐type mechanism.^20^ It can be influenced by four catalytic strategies:^21^ (α) orientation of the 2′ oxygen, phosphor, and 5′ oxygen atoms for in‐line nucleophilic attack;^22^ (β) electrostatic compensation of the enhanced negative charge on the nonbridging phosphate oxygens in the transition state; (γ) general base catalysis by removing the proton from the attacking 2′‐OH nucleophile; and (δ) general acid catalysis by donating a proton to the developing negative charge on the 5′ oxygen leaving group (Figure 1). Moreover, we point out that recent experimental and computational evidence suggests that phosphodiester bond cleavage is not necessarily concerted but can proceed in a stepwise manner involving ‘tight’ transition states that are asynchronous.^20^, ^23^ Distinct structural features of how the RNA molds a ribozyme's active site, impact on the reaction pathway and transition states for RNA 2′‐*O*‐transphosphorylation; these features are responsible for the broad range of cleavage rates that are encountered for individual classes of ribozymes.

In the case of protein‐based enzymes, the chemical versatility of amino‐acids with their various nonpolar, charged and uncharged polar side chains strongly contributes to general acid–base and electrostatic catalysis.^19^ For ribozymes, it was therefore initially less clear how their more limited chemical make‐up could affect general acid–base catalysis at neutral pH, especially since ionization of the ribose and nucleobases in isolation only takes place at considerably acidic or basic conditions. The first reports thus suggested ribozymes to rather be metalloenzymes in which the RNA serves to position partially hydrated Mg^2+^ ions as effectors for ribozyme chemistry.^24^, ^25^ However, with the accumulating evidence that many self‐cleaving ribozymes remain functional in the absence of divalent metal cations, the possibility of p*K*_a_‐shifted active site nucleobases participating directly in catalysis appeared, and the general acid–base catalysis is nowadays considered to be probably the major source of rate enhancement in small self‐cleaving ribozymes.^16^, ^26^ Nevertheless, mechanistic insights into all so far discovered ribozymes prove that none of them behaves like the other and that catalysis is likely multifactorial with other processes contributing to overall rate enhancement. Thereby, special care must be taken in differentiation of direct effects of pH and metal ions on ribozyme catalysis and of indirect effects on the assembly and stability of RNA structures.^19^

Recently, the ribozyme field was reinvigorated by ‘comparative genomic analysis’–based identification of novel nucleolytic ribozyme motifs, termed twister,^27^ twister sister,^28^ pistol,^28^ and hatchet.^28^ These discoveries have opened an opportunity to undertake structure‐function studies to expand on our understanding of mechanistic insights associated with self‐cleaving ribozyme‐mediated catalysis. To this end, recent biochemical,^27^, ^28^ structural,^29^, ^30^, ^31^, ^32^ and chemical^32^ investigations on the twister ribozyme have provided insights into the topological constraints contributing to catalysis. These constraints are particularly interesting because of clear distinctions in the active site and the P1 segment of the currently four available structures, leaving room for vivid interpretations that are discussed in this review.

BOX 1 **SMALL SELF‐CLEAVING RIBOZYMES**

With the discovery of the first ribozyme by Tom Cech in 1982 the view of proteins being the sole catalytic molecules in living organism dramatically changed.^7^ Whereas RNA was considered for a long time to be information carrier only, we know today that RNA's functions go well beyond that task. Among functional RNAs, naturally occurring ribozymes are prominent. Most of them catalyze the same reaction, namely RNA strand scission and its reversal, RNA ligation. According to their main function, ribozymes can be divided into two groups:^33^ splicing ribozymes and cleaving ribozymes, whereas the latter can be further divided into trans‐cleaving ribonuclease P,^34^ and small self‐cleaving ribozymes (often referred to as ‘nucleolytic’ ribozymes). Currently, there are nine distinct small self‐cleaving ribozyme classes known, including hairpin,^35^, ^36^, ^37^, ^38^ hammerhead,^39^, ^40^, ^41^, ^42^, ^43^ hepatitis delta virus (HDV) and HDV‐like motifs,^44^, ^45^, ^46^, ^47^ glucosoamine‐6‐phosphate synthase (glmS),^48^, ^49^, ^50^ Neurospora Varkud satellite (VS),^51^, ^52^ twister,^27^ and the recently discovered twister sister,^28^ hatchet,^28^ and pistol^28^ motifs. All of them form active sites by secondary and tertiary structure interactions that appear unique to each single class. Also, all of them complete intramolecular self‐scission by a combination of defined catalytic strategies that are discussed in the main text. Crystal structures are essential for disclosing the active site functional groups that are potentially involved in catalysis (see Table 1); they provide the basis for proposals of the underlying chemical mechanism of phosphodiester cleavage.^55^, ^56^, ^57^, ^58^ Equally important, ribozymes’ genomic distribution promotes the development of novel computation search strategies for the identification of so far hidden classes.^27^, ^28^

## STRUCTURE OF THETWISTER RIBOZYME

### Secondary Structure Model

**Figure 2:**
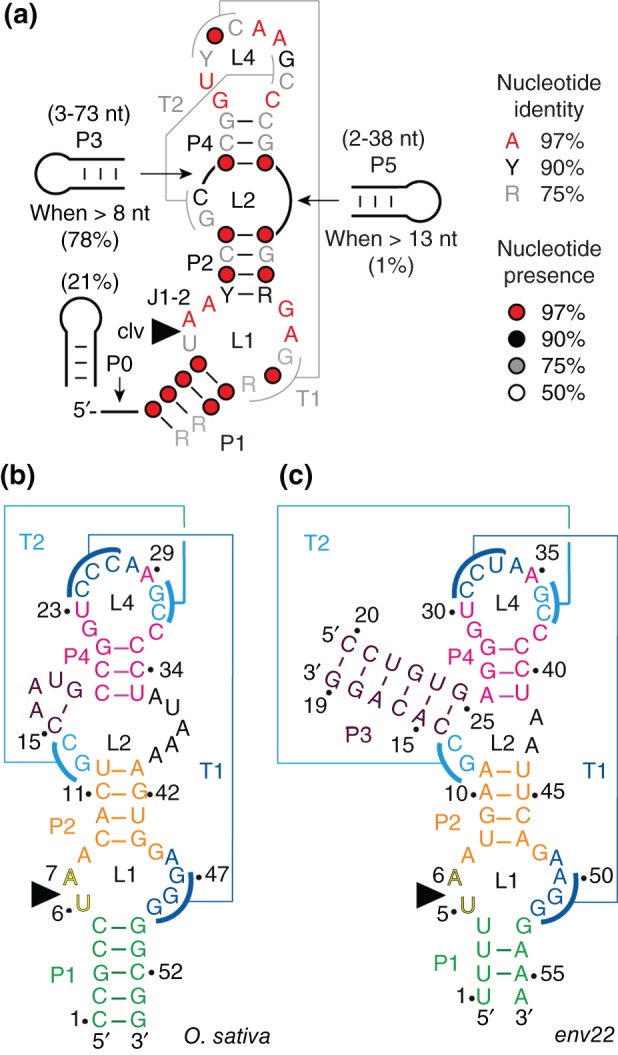
Twister ribozymes. (a) Consensus sequence and secondary structure model for the twister RNA motif (adapted from Refs 27, 28); Watson–Crick base‐paired stems and pseudoknots^59^ are defined by P# and T#, respectively; terminal and internal loops are defined by L#. Detailed sequences of the *O. sativa* (b) and the *env22* (c) twister ribozyme constructs used for structural and biochemical studies.^29^, ^30^, ^31^, ^32^ For the *env9* twister sequence and secondary structure, see Ref 30.

Comparison of more than 2600 twister ribozymes identified in a bioinformatics screen by Roth et al. in early 2014, revealed a detailed consensus secondary structure model for this novel ribozyme class which was further refined recently (Figure 2).^27^, ^28^ Supported by a first set of biochemical in‐line probing experiments, the twister ribozyme was defined to comprise three essential stems (P1, P2, and P4), with up to three additional ones (P0, P3, and P5) of optional occurrence. Since the consensus motif was encountered in circularly permuted RNA versions, three different types of twister ribozymes were assigned depending on whether the termini are located within stem P1 (type P1), stem P3 (type P3), or stem P5 (type P5) (Figure 2(a)).^27^ Such permutations are rather unusual for ribozymes and could thus far only be detected among hammerhead ribozymes.^39^, ^60^ Also in the original paper, the fold of the twister ribozyme was predicted to comprise two pseudoknots^59^ (labeled T1 and T2, respectively), formed by two long‐range tertiary interactions.^27^ Furthermore, phylogenetic analyses revealed at least ten strongly conserved nucleotides (>97% conservation) located within the internal loop L1 and the terminal loop L4, which are brought together through the double‐pseudoknot arrangement. The secondary structure model suggested the active center of the twister ribozyme to be composed of pseudoknot T1 and junction J1‐2, the latter comprising the scissile bond of a highly conserved adenine and a less conserved uridine (5′ to the scissile phosphate) that adjoins to stem P1 (Figure 2).^27^ Consistent with this model, biochemical investigations of mutant constructs revealed decreased cleavage activities upon exchanging one of the conserved nucleotides or upon disruption of the proposed base‐pairing interactions.^27^

Interestingly, a recently described novel class of self‐cleaving ribozymes, named twister sister, shares vague similarities in sequence as well as secondary structure to the twister ribozyme.^28^ However, cleavage occurs at the opposite strand relative to the loop cleaved by twister and there is no evidence of pseudoknot formation. Although both motifs possess a highly, although not invariantly conserved adenine immediately 3′ to the cleavage site, other key conserved nucleotides are not found in the twister‐sister ribozyme. Thus, either both ribozyme classes have distinct active sites that use a similar scaffold, or, although differing in nucleotide composition, both actually form the same geometry and tertiary contacts.^28^

### Comparison of Available Twister Ribozyme Crystal Structures

In order to uncover structural similarities and catalytic strategies used by diverse ribozyme species, atomistic models of any novel functional RNA species are indispensable. Therefore, it is not surprising that three research groups independently published crystal structures (at 2.3, 2.6/2.9, and 3.1/4.1 Å resolution, respectively) of the twister ribozyme shortly after its computational prediction and biochemical verification.^29^, ^30^, ^31^, ^32^ The specific RNAs investigated (sequences either derived from environmental samples^30^, ^31^, ^32^ or from Asian rice, *Oryza sativa* 29, 30) slightly differ in their respective secondary structures (Figure 2). The one published by the Lilley group belongs to a basic P1 type with lacking stems P3 and P5 (Figure 2(b)).^29^ The ones published by the Patel/Micura^31^, ^32^ and the Steitz^30^ groups, respectively, refer to bimolecular constructs excluding stem P5, but including P3 as this stem is present in 78% of annotated twister ribozymes.^27^ The termini of the RNA strands are thereby retained in P1 and P3 (e.g., *env22* twister, Figure 2(c)),^30^, ^31^, ^32^ and therefore these twister ribozymes represent a combination of both type P1 and type P3 motifs.

All crystal structures published thus far trapped the ribozyme in a pre‐catalytic state. To obtain this state, cleavage was prevented by the use of RNAs with a chemically modified cleavage site, carrying either a 2′‐deoxyribose,^29^, ^30^, ^31^ or in the most recent structure,^32^ a 2′‐OCH_3_ modified ribose unit. Furthermore, all available structures adopt a similar global fold, which is consistent with the originally proposed secondary structure model discussed above (Figure 2(a)). As such, the tertiary fold is generated through co‐linear stacking of helical stems and the formation of two pseudoknots and thus represents a continuous A‐form helical architecture. However, there are also clear distinctions among the four reported structures with respect to the active site arrangement and the adjoining stem P1 that will be discussed in more detail below. At this point we also mention, that a fifth crystal structure of the *O. sativa* ribozyme,^30^ is not further included in the discussion, since the crystals contained intermolecular dimer artifacts with a disordered cleavage site.^30^

### Structural Distinctions at the Active Site and the P1 Segment

**Figure 3:**
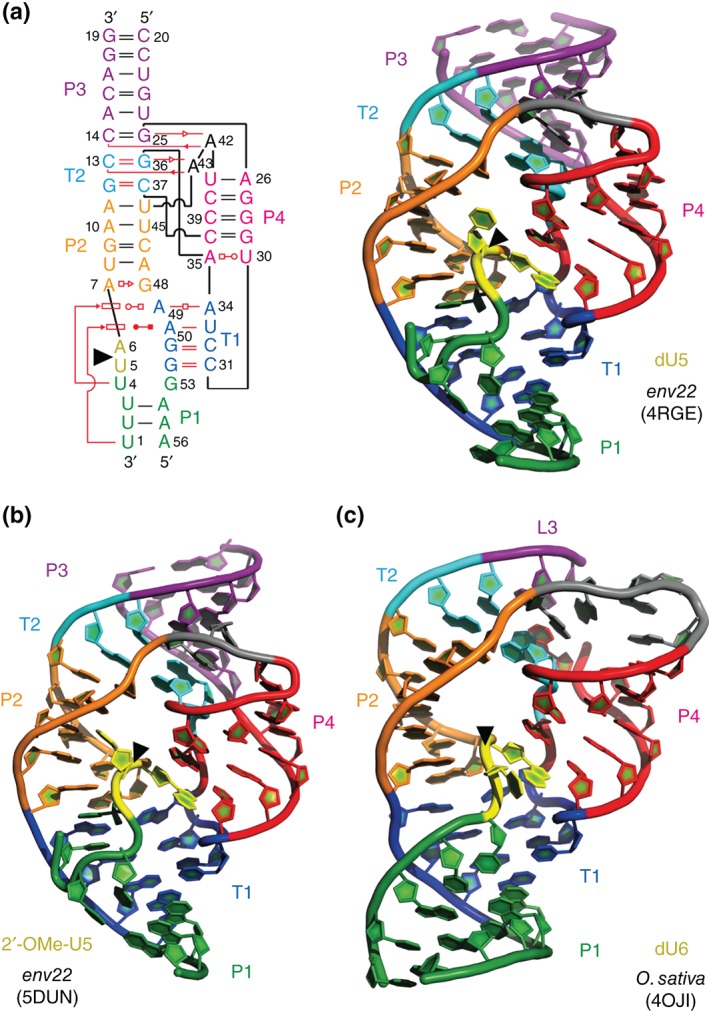
Comparison of *env22* (5DUN and 4RGE), and *O. sativa* (4OJI) twister ribozymes—overall folds. (a) Leontis–Westhof presentation^61^, ^62^ (left) and three‐dimensional structure in cartoon presentation (right) of the dU modified *env22* ribozyme, (b) the 2′‐OCH_3_U modified *env22* ribozyme, and (c) the dU modified *O. sativa* ribozyme. Note the differences in segment P1 (green): ‘back‐folding’ of nucleosides (U1 and U4) of segment P1 for *env22* to form base triplets (also see panel (a)), and fully Watson–Crick base‐paired stem P1 for *O. sativa*, respectively. Black triangles indicate the cleavage positions.

**Figure 4:**
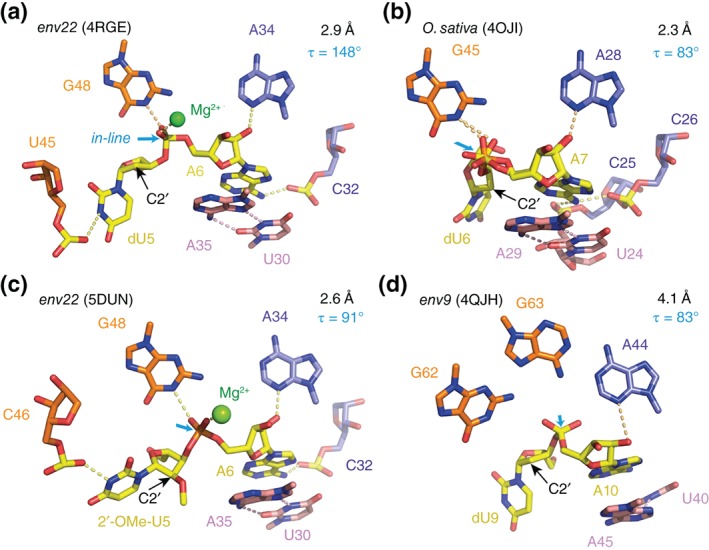
Comparison of *env22* (5DUN and 4RGE), *O. sativa* (4OJI) and *env9* (4QJH) twister ribozymes—active sites and the to‐be‐cleaved dinucleotide units (the latter highlighted in yellow). (a) dU–A arrangement in the 2.9 Å resolution structure of the *env22* twister ribozyme with emphasis on the position of the C2′ of dU relative to the P—O5′ bond (for a presentation with modeled 2′‐OH on C2′, see Ref 31). (b) dU–A arrangement in the 2.3 Å resolution structure of the *O. sativa* twister ribozyme (PDB: 4OJI); local conformational heterogeneity at the scissile phosphate was observed; fitting this to a double conformation (as shown) improved the electron density map (for *F*_o_–*F*_c_ maps, see Figure S3 of Ref 29). (c) 2′‐OCH_3_U–A arrangement in the 2.6 Å resolution structure of the *env22* twister ribozyme (PDB: 5DUN). (d) dU–A arrangement in the 4.1 Å resolution structure of the *env9* twister ribozyme (PDB: 4QJH). Directions for in‐line attack of the O2′ nucleophile at the to‐be‐cleaved P—O5′ bond are indicated by cyan arrows. C2′ positions (where the 2′‐OH nucleophiles are attached in the corresponding functional RNAs) are indicated by black arrows; *τ* describes the angle O2′ (of nucleotide N − 1) to P–O5′ (of nucleotide N + 1) according to Ref 22: in‐line alignment implies a *τ* of 180°.

The first difference is found within the phylogenetically conserved P1 segment, which forms the ‘basement’ of the active site. In the *O. sativa* (2.3 Å, PDB code: 40JI)^29^ and the *env9* twister structure (4.1 Å, PDB code: 4QJH),^30^ all nucleobases of this segment are involved in Watson–Crick base‐pairing interactions and form a classical stem (Figures 2(b) and 1(c) of Ref 30). In contrast, pairing in the P1 segment of the *env22* twister ribozyme (2.9 Å, PDB code: 4RGE; 2.6 Å 5DUN)^31^, ^32^ is restricted to two central Watson–Crick base pairs, whereas the other two nucleotides instead fold back and participate in the formation of base triples (Figures 2(c) and 3(a)). As such, U1 and U4 at either end of segment P1 form stacked U1•(U33•A50) and U4•(A34•A49) major groove base triples that stabilize junctional pairs connecting P1 to pseudoknot stem T1 and stem P2 (Figure 3(a)).^31^ Because of this structural difference of the P1 segments (Figure 3), the question arises, whether preventing formation of these base triples by instead forming a fully base‐paired P1 stem, affects organization of the active site and the arrangement of the (nonconserved) U nucleoside at the U–A cleavage site. Such a scenario has to be kept in mind, since the second major difference between the single crystal structures concerns the alignment of this particular U nucleoside (Figure 4). In the dU5 *env22* structure, the modeled O2′ oxygen of U5 is positioned for near in‐line targeting of the to‐be‐cleaved P—O bond, with a O2′ of U to P‐O5′ distance of 2.8 Å and an angle of 148° (Figure 4(a); 4RGE).^31^ In the *O. sativa* and *env9* structures (that also contained the dU),^29^, ^30^ however, the corresponding modeled O2′ oxygens adopt an orthogonal alignment, with a O2′ of U to P‐O5′ distance of 4.1 and 2.9 Å for the *O. sativa* 29 and the *env9* 30 twister constructs, respectively, and angles of approximately 83° (Figure 4(b) and (d); 4OJI, 4QJH). For the structure of the *O. sativa* ribozyme, the absence of an in‐line alignment was further analyzed.^29^ The authors demonstrated by a computational approach that a local rotation of the U at the U—A cleavage site is possible (without affecting the overall local structure too much) and thereby can position its O2′ for in‐line attack.^29^

The third major difference of the structures concerns the presence/absence of a divalent cation at the cleavage site. The *env22* twister ribozyme revealed one Mg^2+^ ion directly coordinated to the pro‐*S* oxygen of the scissile phosphate and additionally coordinated to one of the nonbridging oxygens (pro‐*R*) of the successive downstream phosphate group (Figure 4(a); 4RGE).^31^ This is in contrast to the other two crystal structures where such an interaction of a divalent cation at the cleavage site was not observed. The reason for the ‘missing’ Mg^2+^ in the *O. sativa* ribozyme might be associated with the orthogonal alignment of the dU nucleoside observed in this structure. A detailed look at the local environment surrounding the *O. sativa* cleavage site, however, shows that a Mg^2+^ ion is located in about 5.5 Å distance to the nonbridging oxygens of the scissile phosphate (Figure 4(b); 4OJI),^29^ a position from which it might approach to capture the rotated in‐line aligned arrangement of the U nucleoside in the functional complex. This Mg^2+^ ion is innersphere coordinated to the neighboring phosphate (pro‐*S* nonbridging oxygen) upstream to the scissile phosphate (see also Figure S7 in Ref 29).

The latest crystal structure of the twister ribozyme that has been solved also emphasizes the relevance of the metal ion at the binding site.^32^ This *env22* ribozyme structure at 2.6 Å resolution (Figure 4(c); 5DUN) is of particular interest because it captured a nonlinear (orthogonally aligned) conformation of the U5—A6 cleavage site dinucleotide (~90°), but with Mg^2+^ still coordinated to the scissile phosphate (Mg^2+^ to O distances in the 2.1 Å range) (Figure 4(c); 5DUN),^32^ a situation that was not seen in the other suboptimally aligned structures of twister ribozymes (4QJH, 40JI), including the one at highest resolution (2.3 Å, 40JI).^29^, ^30^ Importantly, the structure was obtained with 2′‐OCH_3_ uridine at the cleavage site instead of 2′‐deoxyuridine and the U5 ribose pucker was changed from C3′‐endo for the in‐line aligned dU5 *env22* ribozyme (4RGE) into C2′‐endo for the orthogonally aligned 2′‐OCH_3_‐U5 *env22* ribozyme (5DUN). We speculate that the altered sugar pucker and the rotation into a suboptimal alignment of 2′‐OCH_3_‐U5 might be a result of steric interference of the bulky 2′‐OCH_3_ group. Otherwise, we note that the P1 segment with its base triplets and only two Watson‐Crick base pairs was similar to the in‐line aligned structure of the *env22* twister ribozyme.^31^, ^32^

### Similarities in the Available Crystal Structures

Moving from the P1 segment and the internal loop/pseudoknot T1 region, further up on the secondary structure model (Figure 2(a)), stem P2 is encountered. As proposed by bioinformatics, this stem consists of four Watson–Crick base pairs. However, all four crystal structures unveil that the stem is extended by the formation of a sheared *trans*‐Hoogsteen sugar edge A•G pair, which is located coaxially in between T1 and P2 (Figures 2(b), 2(c), and 3(a)). It was suggested that any addition or deletion in the number of base pairs of stem P2 might disrupt or interfere with the formation of the active site.^30^

Stem P3 is phylogenetically expendable and in about 20% of currently annotated twister RNAs replaced by a short junction, as seen in the *O. sativa* twister structure.^27^, ^29^ Nevertheless, all of the crystal structures are uniform and consistent with the proposed secondary structure model in respect to the anchorage of stem P3. Importantly, the dinucleotide insertion (conserved GC) within loop L2 (Figures 2 and 3) determines pseudoknot T2 formation that becomes coaxially stacked between P2 and P3.

The P4 segment was bioinformatically predicted to form a three‐base pair stem.^27^ All four crystal structures however amended this prediction, by showing that stem P4 is extended by pairing of nucleotides originally assigned to the L4 loop; these interactions are an additional and essentially invariant G—C Watson–Crick base pair, as well as a *trans* Watson—Crick—Hoogsteen pair formed between a conserved U and A (Figure 3). This arrangement in turn causes the two nucleotides embedded between the newly found base pairs to bulge out and to become available for pseudoknot T2 formation (Figure 3). The complementary half of this pseudoknot resides in L2 (conserved GC) as mentioned before.

The remaining four nucleotides of the terminal loop L4 participate in formation of a second long‐range tertiary interaction with nucleotides from the internal loop L1, thereby generating pseudoknot T1 (Figure 2). T1 involves three Watson—Crick base pairs as predicted and an unanticipated noncanonical A—A *trans*‐Watson—Crick base pair (Figure 3(a)). These tertiary interactions mold loop L4 as well as stem P4 as part of the active site. Consequently, the L4‐P4/L1‐T1 architecture can be considered as key for an active ribozyme structure (Figure 3).

Taken together, all four twister structures adopt a similar global fold stabilized by the same dual pseudoknots and a continuous series of coaxial stems. They, however, differ in the positioning of key residues (in particular, the U at the cleavage site) and the presence/absence of a divalent cation at the cleavage site. These differences likely hint at the intrinsic conformational flexibility that is required for this ribozyme to function, with the individual crystal structures representing snapshots of important conformations on the way to cleavage. The obvious rotational freedom of the nucleoside 5′ to the scissile phosphate is pointed out. This nucleoside makes hardly any contacts with the active site. Its base is directed outward and its solvent‐accessible area is significantly larger compared to other ribozymes’ nucleosides that are 5′ to the scissile phosphate (Table 1). From this course, access of metal ions for (transient) interaction with the scissile phosphate is warranted.

**Table 1:** Catalytic Contributions to Self‐Cleavage Proposed for Selected Ribozyme Classes^1^

Ribozyme Class	α*‐*Catalysis	β*‐*Catalysis	γ‐Catalysis	δ‐Catalysis	Cleavage Rate^3^ *k*_obs_(min^−1^)	References	Solvent Accessibility of N − 1 at Cleavage Site^4^ Area (Å^2^)
	Alignment^2^	Stabilization of Transition State	2′‐OH Activation; General Base Catalysis	5′‐O Activation; General Acid Catalysis			
Twister *env22*	148° (4RGE) 91° (5DUN)	Mg^2+^, G48	G48	A6	2.44 ± 0.31^5^ 1.41 ± 0.16^6^	31, 32	178 (dU5) 189 (2′OMeU5)
Twister *O. sativa*	83° (4OJI)	G45	G45	A7	2.45 ± 0.04	29	—
Twister *env9*	83° (4QJH)	A63	G62	—	ND	30	—
Pistol *env25*	167° (5K7C)	—	G40	A32	2.72 ± 0.38^5^ 0.88 ± 0.07^6^	53	112 (dG53)
RzB Hammerhead	140° (5DI2)	—	G12	G8, Mn^2+^–OH_2_	1.39 ± 0.04	40, 43	78 (dC17)
Hairpin	—	G8	G8	A38	0.1–0.5	37, 54	—
HDV	—	Mg^2+^	Mg^2+^	C75	52	45	—
*Neurospora* VS	—	G638	G638	A756	1	51	—
*glmS*	—	GlcN6P	G40	GlcN6P	1–3	49	—

ND, not determined.

^1^ Self‐cleavage by nucleolytic ribozymes can be influenced by four catalytic strategies (see main text); nucleoside numbering refers to the individual RNA sequences as reported in the (original) papers (second column ‘References’ from the right).

^2^ Definition of τ: angle of O2′ (of nucleotide N + 1) to P–O5′ (of nucleotide N − 1), see Ref 22; for ideal in‐line alignment τ corresponds to 180°.

^3^ Measured at neutral pH.

^4^ Calculated by *areaimol—CCP4*.

^5^ Measured at 20°C.

^6^ Measured at 15°C.

## INSIGHTS INTO THE CATALYTIC MECHANISM OF THETWISTER RIBOZYME

Like most other small nucleolytic ribozymes, the twister ribozyme appears to follow the S_N_2‐type mechanism for phosphodiester cleavage, under the assistance of active site functional groups that have the potential for successful proton transfer (such as 2′‐hydroxyl groups, nucleobases, hydrated metal ions, and phosphate nonbridging oxygen atoms). Investigations of the cleavage kinetics revealed rates in the range of 1–10 min^−1^ with a strong dependence on pH value, Mg^2+^ ion concentration, and temperature.^27^, ^29^, ^31^ The bell‐shaped pH dependence of the cleavage rate can be interpreted in terms of two ionization events (corresponding p*K*_a_ values of 6.9 and 9.5, respectively) and thus would be consistent with transfer of two protons in the transition state of a concerted, general acid–base catalyzed reaction.^29^

Although all four crystal structures of the twister ribozyme differ to some extent in positioning of active site residues, as discussed above, all emphasize a key role for the same invariant guanine (Figure 4) in the cleavage mechanism. In the *env22* twister structure,^31^ where the O2′ modeled onto dU at the cleavage site is properly positioned for in‐line attack to cleave the P—O5′ bond, the N1‐H of this guanine forms a hydrogen bond to the pro‐*S*_P_ nonbridging scissile phosphate oxygen (2.3 Å distance N‐to‐O) and hence argues in favor of a stabilizing role of the transition state during catalysis (ß catalysis) (Figure 1 and Table 1).^31^ Relating to the *env9* 30 and *O. sativa* 29 structures, this guanine was discussed to activate/deprotonate the nucleophile for attack at the phosphorus center. It likely accounts for the p*K*_a_ value of 9.5 and may act as general base during catalysis (γ catalysis) (Figure 1 and Table 1),^29^, ^30^ although the distance between the N1 to the C2′ is rather far (5.3 Å) in the *env9* twister structure (Figure 4(a));^30^ likewise, in the *O. sativa* structure, the N1 becomes juxtaposed with the O2′ nucleophile only after modeling a local rotation of the uridine at the cleavage site (Figure 4(b)).^29^ In this context, Lilley and coworkers state that their pH *versus* rate kinetic data are consistent with the guanine acting as either general acid or base in the cleavage reaction but can neither prove nor disprove the deprotonation model.^29^ Consequently, the contribution of the highly conserved guanine to catalysis remains elusive and requires further investigations.

Turning the attention to the second ionization event (p*K*_a_ ~6.5) implicated by the observed pH‐dependence of cleavage rates,^29^ the question arises which functional group is available and whether this group is located properly to contribute to general acid catalysis (δ catalysis) (Figure 1 and Table 1), by donating a proton to the developing negative charge on the 5′ oxygen leaving group. Two contributing units are conceivable, the adenine at the cleavage site and a hydrated Mg^2+^ ion coordinated to the pro‐*S* oxygen of the scissile phosphate (as seen in two of the four available crystal structures; Figure 4(a) and (c)). Experimental evidence for the adenine to act as general acid stems from the NMR spectroscopically determined p*K*_a_ of 5.1 using a twister ribozyme with a single ^13^C reporter atom in direct neighborhood to the putative protonation site (^13^C2 of adenine).^32^ The p*K*_a_ value was shifted by 1.4 units in comparison to the substrate strand measured alone. The second line of evidence comes from the substitution of this adenine by 1,3‐dideaza‐adenine, which made the ribozyme completely inactive.^31^ It is known that the major protonation site of free adenosine is N1 (96%), followed by N7 (3.2%), and N7/N3 (0.7%).^63^ Although in the twister ribozyme only the adenine N3 (and not the N1) position comes reasonably close to C5′ (3.9 Å in *env22*),^31^ it is tempting to propose that N3 is crucially involved in the cleavage mechanism. Our very recent observation of a 3‐deazaadenosine substituted twister ribozyme that has no activity is consistent with an involvement of N3 in proton transfer (S. Neuner, R. Micura, unpublished results). In this context, we furthermore point at a study on the mechanism of RNA phosphodiester cleavage in the gas phase that assigned a crucial role of intramolecular hydrogen bonding of the (protonated) phosphate backbone units to the N3 of adenine and guanine bases.^64^

Beside the potential role of adenine as a general acid in the twister ribozyme, the impact of divalent metal ions on phosphodiester cleavage cannot be neglected. Earlier biochemical studies on the twister ribozyme did not suggest a direct participation of divalent metal ions in the cleavage mechanism and assigned them a structural rather than a catalytical role.^27^ However, as described above, in both (in‐line and orthogonally aligned) *env22* twister structures,^31^, ^32^ a Mg^2+^ ion is coordinated directly to one of the nonbridging phosphate oxygen at the U–A cleavage site dinucleotide as well as to one of the nonbridging phosphate oxygens of the following A–A sequence step. This Mg^2+^ ion can contribute to electrostatic stabilization of the developing negative charge upon phosphodiester cleavage (Table 1).^31^, ^32^ Moreover, one of the coordinated water molecules is ideally positioned to donate its proton to the O5′ leaving group.^31^

As mentioned above, in the *O. sativa* structure a Mg^2+^ ion was identified to be innersphere coordinated to the phosphate of the C‐U dinucleotide unit preceding the cleavage site.^29^ It is only in about 5.5 Å distance to the scissile phosphate, a position from which it could easily approach and complex the in‐line aligned arrangement of the U nucleoside in the dynamic, functional complex.

The available twister crystal structures are consistent with the notion of high conformational flexibility encountered for the nonconserved U at the cleavage site, while the two candidates for general acid and base catalysis (A and G; Table 1) of the active site are more comparably arranged. The uridine has been captured in differently rotated conformations ranging from orthogonal to close to in‐line alignment (Figures 3 and 4). This implies that the energetic barrier for the rotation is low and that the various local conformers can be easily captured, e.g., by a single hydrogen bond interaction (H‐bond of N3–H U5 to the phosphate of the U44–U45 sequence unit in 4RGE (Figure 4(a));^31^ H‐bond of N3–H U5 to phosphate of the U45–C46 sequence unit in 5DUN (Figure 4(c))),^32^ or through crystal packing effects (H‐bond of N2–H_2_ G23 to O4–U6′ of the symmetry‐related ribozyme; see Figure S9 in Ref 29).

**Figure 5:**
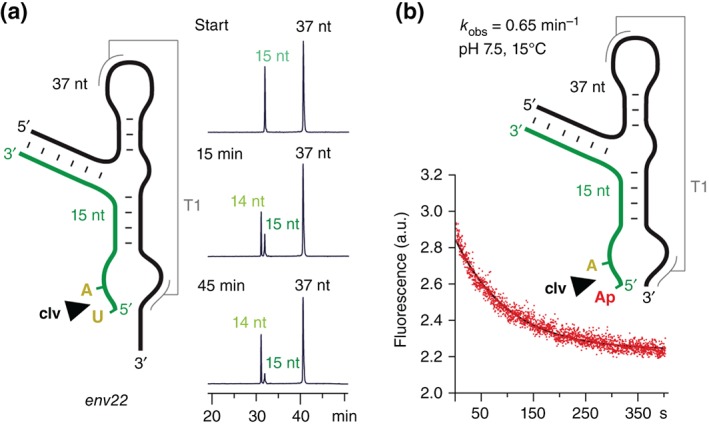
Single nucleotide cleavage of the twister ribozyme. (a) The twister ribozyme does not require formation of the phylogenetically conserved stem P1 for efficient cleavage.^32^ Exemplary HPLC cleavage assay for a 5′‐truncated substrate RNA, showing that a single nucleotide (U5) is cleaved. Conditions: 2 mm MgCl_2_, 100 mm KCl, 30 mm HEPES, pH 7.5, 23°C. (b) Cleavage kinetics of a twister ribozyme lacking stem P1 (‘mini‐twister’) analyzed by ensemble 2‐aminopurine (Ap) fluorescence spectroscopy. The nonconserved U5 was replaced by Ap (in red letters). Note that Ap fluorescence decreases during the course of cleavage; this observation hints at the very exposed and unshielded arrangement that the nucleobase has to adopt (active conformation) to become cleaved; conditions: c_RNA_ = 0.3 μM, 50 mM potassium 3‐(*N*‐morpholino)propanesulfonate (KMOPS), 100 mM KCl, 15°C, pH 7.5; mixing was performed manually in less than 2 seconds resulting in 10 mM Mg^2+^ concentration (see also Figure S2 in Ref 32).

It is important to note that stem P1, although phylogenetically conserved, is dispensable for cleavage activity. Twister ribozymes that lack stem P1 cleave a single nucleoside with almost wild‐type rate (Figure 5).^32^ Unfortunately, our attempts to crystallize a twister ribozyme that lacks stem P1 failed so far. In this context, we are wondering if the differences seen between the *O. sativa* and the two *env22* active sites with respect to the locations of the Mg^2+^ ion might propagate from the different P1 arrangements (discussed above) captured in the *O. sativa* 31 versus *env22* 32 crystals. More straightforward to rationalize, appears the different orientation of the uridine in the orthogonally aligned *env22* ribozyme^32^ compared to the in‐line aligned *env22* ribozyme.^31^ This difference likely arises from the dU *versus* (bulkier) 2′‐OCH_3_‐U substitution at the cleavage site (Figure 4(a) and (c)).

Finally, we point out that additional experimental evidence for the involvement of divalent metal ions in catalysis originates from activity assays that were performed using diastereomerically pure phosphorothioate RNA substrates.^32^ Cleavage of the *S*_P_ diastereomer is accelerated in the presence of thiophilic metal ions such as Mn^2+^ or Cd^2+^. In contrast, the *R*_P_ diastereoisomer is not cleaved under the same conditions. Most likely, the hydrogen bond of the active site guanine to the sulfur atom of the *R*_P_ phosphorothioate substrate is much weaker, and consequently, its proper positioning toward the guanine N1 is impaired. In general, the observed thio/rescue effects were small but significant,^32^ and the data obtained are consistent with the notion of ‘cleavage assistance’ through divalent metal ions that are transiently bound to the scissile phosphate. The thio/rescue effect on twister ribozymes was recently re‐confirmed independently.^65^

Another very recent study,^66^ a computational analysis, pointed at another metal ion, a Na^+^ ion that remained close to A(O5′), the leaving group atom, during the majority of the microsecond trajectories, suggesting that it might stabilize the negative charge on A(O5′) during self‐cleavage. The location of this cation in the active site suggests that it may be catalytically relevant as well.

**Figure 6:**
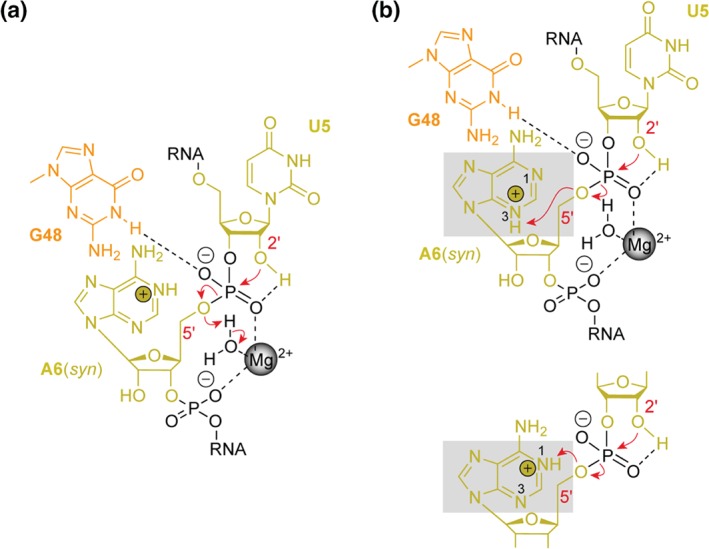
Mechanistic proposal for phosphodiester cleavage in the twister ribozyme, exemplified for the *env22* RNA. (a) Guanine‐48 can stabilize the transition state and may also be involved in activation of the U5 2′‐OH. The Mg^2+^ ion coordinated to the scissile phosphate (and additionally clamped to the successive phosphate unit) also stabilizes the transition state and may assist to neutralize the developing charge on the leaving O5′ through donation of a proton *via* a coordinated water molecule. (b) A further candidate for acting as general acid is adenine‐6. This adenine possesses a shifted p*K*_a_ of 5.1 as determined by NMR spectroscopy,^32^ and may donate its proton to the leaving O5′. From a structural perspective, however, only N3 is appropriately positioned in vicinity to O5′ (top), and not N1 (bottom inset); the latter representing the preferred protonation site of a protonated adenine (>96% versus 0.7% for N7/N3 according to reference 63). Additional studies are required to complete our understanding of the mechanism for twister ribozymes that most likely use a combination of catalytic strategies as discussed in the main text.

Altogether, data from chemical, biochemical, and structural experiments support the idea that the twister ribozyme uses a combination of strategies in order to attain its high cleavage rate (Figure 6 and Table 1). First of all, twister employs classic ‘α catalysis’, which results from forming the active site (with pseudoknot T2 as most critical long‐range tertiary interaction) and thereby allowing orientation of the to‐be‐cleaved P—O bond for in‐line nucleophilic attack. The observed pH dependence additionally is in favor of ‘γ catalysis’, with the conserved guanine representing a potential candidate to act as general base. Also, a role in stabilizing the transition state can be attributed to the same guanine (as seen for other nucleolytic ribozymes; Table 1)^8^. For general acid catalysis (δ), the adenine base directly at the cleavage site is an attractive candidate although its contribution in proton transfer likely involves N3 and not N1, for reason of vicinity. Importantly, transient coordination of Mg^2+^ to the scissile phosphate (ß catalysis) can also contribute to rate acceleration of the twister ribozyme by stabilizing the transition state and likely by directly donating a proton of a Mg^2+^‐coordinated water molecule to the O5′ leaving group (Figure 6).

**Figure 7:**
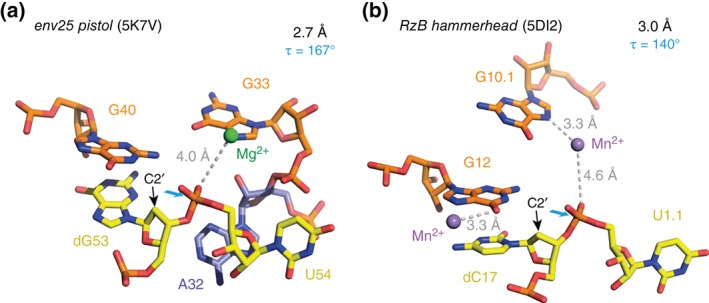
Comparison to pistol and hammerhead ribozymes—active sites and the to‐be‐cleaved dinucleotide units (the latter highlighted in yellow). (a) dG–U arrangement in the 2.7 Å resolution structure of the *env25* pistol ribozyme (PDB: 5K7V) with emphasis on the position of the C2′ of dG relative to the P—O5′ bond (for a presentation with modeled 2′‐OH on C2′, see Ref 53). (b) dC–U arrangement in the 3.0 Å resolution structure of the *RzB* hammerhead ribozyme (PDB: 5DI2).^40^ Directions for in‐line attack of the O2′ nucleophile at the to‐be‐cleaved P—O5′ bond are indicated by cyan arrows. C2′ positions (where the 2′‐OH nucleophiles are attached in the corresponding functional RNAs) are indicated by black arrows; *τ* describes the angle O2′ (of nucleotide N − 1) to P–O5′ (of nucleotide N + 1) according to reference 22: in‐line alignment implies a *τ* of 180°.

The most recently disclosed structure of a ribozyme refers to the pistol motive.^28^ It displays a compact architecture with an embedded pseudoknot (Figure 7(a)).^53^ Its G–U cleavage site adopts a splayed‐apart conformation with in‐line alignment of the 2′‐O nucleophile ready for attack on the to‐be‐cleaved P—O5′ bond. The N1 position of a highly conserved guanine (G40) is properly positioned to act as a general base while the N3 and 2′‐OH positions of an adenine (A32) are located to be candidates for general acid catalysis (Table 1). Both nucleotides play a significant role in cleavage rate acceleration. We experimentally determined a p*K*_a_ of 4.7 for A32 (increased by one p*K*_a_ unit when compared to adenine in a single‐ or double‐stranded environment).^53^ Interestingly, the A32G pistol ribozyme mutant showed activity comparable to wild‐type. Together, these findings suggest that the purine‐32 N3 is of crucial importance for cleavage activity.^53^ The nonbridging oxygens of pistol scissile phosphate did not showed inner‐sphere coordination to a Mg^2+^ ion, a situation distinct to the one found for twister ribozymes. The pistol ribozyme rather shows a Mg^2+^ coordinated to N7 of a guanine (G33) and in 4 Å distance to the nonbridging oxygens of the scissile phosphate. This situation is reminiscent to the RzB hammerhead ribozyme (at pH 8) that was reported recently (5DI2)^40^ and that also displayed metal ion coordination (Mn^2+^) to N7 of a guanine (G10.1) and a distance of 4.6 Å to the nonbridging oxygens of its scissile phosphate (Figure 7(b)).

## BIOLOGICAL RELEVANCE AND POTENTIAL APPLICATION OF THE TWISTER RIBOZYME

Although the discovery of ribozymes has been fundamental for our understanding of the biochemical, structural, and biological versatility of functional RNA, and thus to the epistemological construction of the RNA world hypothesis, their functional role in the living organisms remains elusive.^12^ Only for selected systems, a more detailed knowledge has been attained. For instance, there is profound evidence that the *glmS* ribozyme is unique in its role as metabolite‐responsive self‐cleaving ribozyme, acting as riboswitch that regulates gene expression in many Gram‐positive bacteria.^67^ Furthermore, the VS and hairpin ribozymes are associated with defined biological roles in viral genome replication,^16^, ^68^ HDV ribozymes catalyze their own scission from the transcript during rolling circle replication of the hepatitis delta virus, and HDV‐like ribozymes are being part of the life cycle of a certain class of transposons.^69^ Less clear is the situation for hammerhead and twister ribozymes that are comparably widespread in nature with thousands of representatives in living systems; their biological functions are currently mainly linked to their mere genomic context and thus remain largely speculative.^12^ Since many twister and hammerhead RNAs commonly locate within a few kilobases of each other and likewise near specific protein‐coding genes,^27^ a potential role during pre‐mRNA biosynthesis may be among others. Very recently, the twister ribozyme, as well as other structured functional RNAs, could additionally be linked to the activation of the human innate immune system.^70^ Therefore, twister like other ribozyme classes, possesses potential to be engineered into therapeutic agents, targeting specific (pathogenic) RNAs. More facile to realize, twister can serve as flexible module for the engineering of biosensors and hence contributes to enrich the synthetic biologist's toolbox.^71^, ^72^ In this context, especially the merging of ribozymes and riboswitches to so‐called aptazymes has gained much attention in the field of functional genomics and gene research.^73^ Thereby, the ribozyme represents the expression platform of the aptazyme that adjoins the aptamer domain (the molecular recognition motif), usually through partial sequence overlap. Depending on the concentration of the small molecule that specifically binds to the aptamer, the ribozyme, in turn, adopts the active fold to allow cleavage (usually in *cis*). The dose‐dependent ‘signal’ is most commonly embedded in an ‘off‐regulatory’ event. Twister is considered as promising candidate for this engineering design due to several reasons.^71^ First, twister is among the fastest cleaving ribozymes.^27^, ^28^ Second, since the twister motif is present in both bacteria and eukaryotes, it can be virtually used for applications in diverse organisms.^27^ Furthermore, twister offers several strategic sites for modifications, enabling the connection to the aptamer domain. Altogether, the versatility of the twister motif together with its catalytic efficiency assures the future of its application to be broad.

## CONCLUSION

Four crystal structures of the twister ribozyme with ordered active sites together with a series of chemical and biochemical experiments provide insights into how the twister ribozyme accomplishes its efficient phosphodiester cleavage. The highly conserved guanine and adenine at the cleavage site appear to be the important contributors to cleavage chemistry, likely being involved in general acid base catalysis. Moreover, evidence for innersphere coordination of Mg^2+^ to one of the nonbridging oxygens of the scissile phosphate stems from two of the crystal structures. Together with the finding of thio/rescue effects for phosphorothioate substrates, this suggests the participation of divalent ions in the overall catalytic strategy employed by twister ribozymes, an aspect that has been unfortunately overlooked in a recent review^74^ and a computational study on this ribozyme class.^75^

Our current efforts toward a comprehensive mechanistic understanding of the twister ribozyme focus on single‐molecule two‐ and three‐color FRET experiments with the aim to shed light on the interdependencies between ribozyme substrate annealing and ribozyme folding and cleavage.
